# A Misleading Cystic Brain Lesion: An Uncommon Presentation of Meningioma

**DOI:** 10.7759/cureus.104850

**Published:** 2026-03-08

**Authors:** Alpha Singuepire, Yelka Matos Furones, Elizabeth Blanco Espinosa, Idania Cruzata Matos, Luis F Gonzalez Vazquez, Julio C Matos Cruzata

**Affiliations:** 1 Neurological Surgery, Hôpital Nianankoro Fomba de Segou, Segou, MLI; 2 North Georgia Clinical Research/Neurology, Alcanza Clinical Research, Woodstock, USA; 3 General Practice, CEDA Orthopedic Group, Miami, USA; 4 General Surgery, Hospital Universitario Arnaldo Milian Castro, Santa Clara, CUB; 5 General Medicine, HCA Healthcare, Las Vegas, USA; 6 Anesthesiology, Geisinger Medical Center, Danville, USA; 7 Family Medicine, Hospital Provincial Saturnino Lora, Santiago de Cuba, CUB

**Keywords:** cystic meningioma, diagnostic challenge, intracranial tumors, meningothelial meningioma, surgical management

## Abstract

Cystic meningiomas are an uncommon presentation that may pose diagnostic challenges due to their atypical radiological features, frequently mimicking other cystic intracranial neoplasms.

We report the case of a 40-year-old woman who presented with recurrent generalized tonic-clonic seizures, beginning in late pregnancy and the postpartum period, followed by progressive headaches and urinary incontinence. Neurological examination revealed psychomotor slowing, right-sided hemiparesis, and signs consistent with an upper motor neuron lesion. Neuroimaging demonstrated a well-circumscribed left frontoparietal extra-axial mass with a predominant cystic component, an enhancing mural nodule, significant mass effect, and midline shift, initially suggestive of a glial tumor. The patient underwent gross total surgical resection. Histopathological analysis confirmed a meningothelial meningioma with cystic features. Postoperatively, the patient showed clinical improvement without new neurological deficits.

This case highlights the importance of considering cystic meningioma in the differential diagnosis of cystic brain lesions, particularly when imaging findings are atypical. Early recognition and appropriate surgical management are essential to achieve favorable clinical outcomes.

## Introduction

Meningiomas are the most common primary tumors of the central nervous system, accounting for approximately 30%-40% of all primary brain tumors worldwide. The annual incidence is approximately 7-9 cases per 100,000 person-years and shows a clear female predominance, particularly in benign (WHO grade I) meningiomas. Incidence increases with age, and the widespread use of modern neuroimaging has led to an increasing number of incidentally detected cases. These epidemiological features establish meningiomas as a major contributor to the global burden of intracranial neoplasms [1-6].

Cystic meningiomas represent a rare and diagnostically challenging variant, accounting for approximately 4%-7% of all intracranial meningiomas. The presence of a cystic component can significantly complicate the radiological diagnosis, as these lesions are frequently mistaken for other intra-axial tumors, such as gliomas, metastases, hemangioblastomas, or pilocytic astrocytomas, due to their imaging characteristics and contrast-enhancing mural nodules [7,8].

Both purely cystic and mixed cystic-solid meningiomas have been described, with cysts classified as intratumoral or peritumoral, although this classification does not consistently correlate with a specific histological subtype or WHO grade [9,10]. Despite their atypical radiological features, most cystic meningiomas are histologically benign (WHO grade I) and demonstrate favorable outcomes when complete surgical resection is achieved, including removal of the cyst wall when indicated [7,11].

Because their imaging appearance may closely mimic intra-axial neoplasms, cystic meningiomas remain an important diagnostic pitfall in neuroimaging and neurosurgical practice. Accurate recognition of this entity is essential for appropriate surgical planning and management.

In this report, we present a case of cystic meningioma in a female patient treated surgically at our institution, highlighting the diagnostic challenges associated with this rare variant and emphasizing the importance of considering cystic meningioma in the differential diagnosis of cystic intracranial lesions.

## Case presentation

History and clinical findings

A 40-year-old woman with no prior medical history and no known family history of epilepsy or intracranial tumors presented with a history of tonic-clonic seizures. The first seizure occurred approximately two years earlier, during the third trimester of pregnancy (without associated preeclampsia, eclampsia, or metabolic disturbances), and was managed in a Gynecology Department without brain imaging. One month postpartum, she experienced a second tonic-clonic seizure, after which antiepileptic medication was prescribed without further etiological investigation. Approximately two months prior to admission to the Neurosurgery Department, the patient developed moderate headaches that progressively worsened and were associated with urinary incontinence. The headaches were described as diffuse, non-pulsatile, and more intense in the morning, suggestive of raised intracranial pressure. There was no associated fever, visual aura, or focal sensory complaints. She was initially evaluated by a urology service; however, following a non-contrast and contrast brain computed tomography (CT), she was referred to Neurosurgery for further management.

Neurological examination

On neurological examination at admission, the patient exhibited psychomotor slowing and behavioral disturbances. She was oriented to person but partially disoriented to time, with reduced verbal fluency and impaired attention span. Cranial nerve examination revealed no facial asymmetry, normal pupillary reflexes, and intact extraocular movements. Fundoscopic examination was not contributory. Motor examination revealed right-sided hemiparesis, graded at 3/5 according to the Medical Research Council scale [12]. The weakness predominantly involved the upper limb, with decreased fine motor coordination and a positive pronator drift. Deep tendon reflexes were brisk on the right side, with a positive Babinski sign consistent with an upper motor neuron lesion. Muscle tone was mildly increased on the affected side. The Glasgow Coma Scale score was 14. Urinary incontinence was present, and assessment of sensory function was limited due to poor cooperation. Gait assessment was not feasible due to motor deficit and reduced cooperation. The overall findings were consistent with a left hemispheric extra-axial expansive lesion, causing mass effect and corticospinal tract compression.

Neuroimaging findings

**Figure 1:**
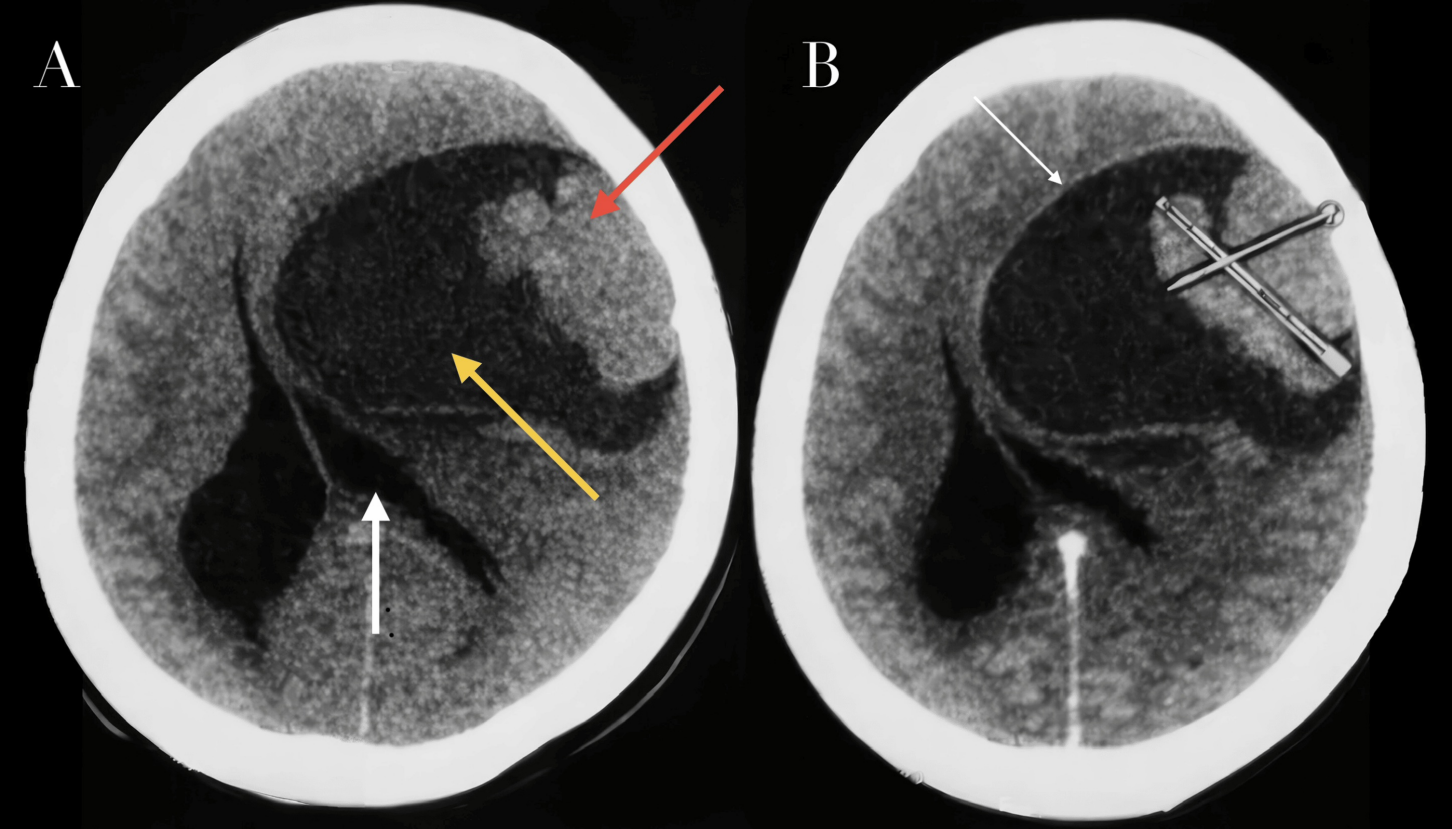
Axial non-contrast brain CT (A) and contrast CT (B) images demonstrate a well-defined extra-axial mass along the cerebral convexity. (A) Non-contrast axial CT demonstrates a large extra-axial lesion with both solid (red arrow) and cystic (yellow arrow) components, causing significant mass effect, with midline shift and near-complete compression of the ipsilateral lateral ventricle (white arrow). (B) Contrast-enhanced axial CT shows marked enhancement of the solid component, and enhancement of the cyst wall (white arrow), further supporting the diagnosis of a dural-based, extra-axial tumor. CT, computed tomography

**Figure 2:**
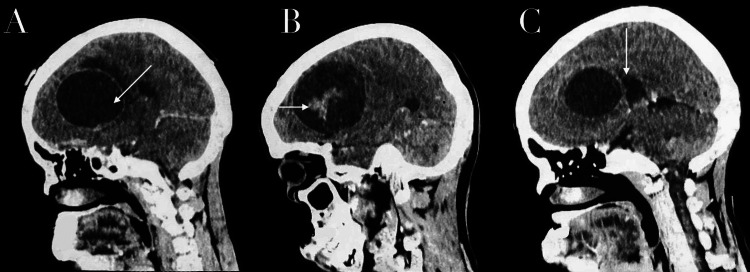
Contrast-enhanced sagittal CT images of the brain. (A) Sagittal contrast-enhanced CT reconstruction demonstrates a well-defined frontoparietal cystic lesion (white arrow) with ring enhancement. (B) Paramedian sagittal post-contrast CT image demonstrates a hyperdense focus within the cystic cavity, corresponding to the solid component of the lesion (white arrow). (C) More medial sagittal post-contrast CT image illustrates displacement of the lateral ventricle by the tumor (white arrow), which is located anterior to the ventricular system. CT, computed tomography

Neuroimaging demonstrated a large frontoparietal lesion with mixed cystic and solid components. The lesion produced a significant mass effect, characterized by midline shift and near-complete compression of the ipsilateral lateral ventricle. A broad-based dural attachment was identified on axial imaging, supporting an extra-axial origin (Figure 1A). Following intravenous contrast administration, there was marked enhancement of the solid tumor component, as well as enhancement of the cyst wall, supporting the presence of a neoplastic cystic lesion rather than a simple cystic structure (Figure 1B). Sagittal CT reconstructions confirmed the presence of a well-defined cystic lesion with an internal solid component and peripheral capsular enhancement. In the sagittal plane, the lesion was visualized anterior to the lateral ventricle. Overall, the imaging findings were consistent with a predominantly extra-axial frontoparietal mass with cystic and solid components, radiographically suggestive of a cystic meningioma (Figure 2). Magnetic resonance imaging (MRI), which represents the standard imaging modality for the characterization of intracranial tumors, was not performed in this case due to limited institutional availability and the patient’s progressive neurological deterioration, associated with significant mass effect.

Differential diagnosis

Based on the radiological appearance of a well-defined extra-axial mass with a prominent cystic component and significant mass effect, several entities were considered. A hemangiopericytoma (currently classified within the spectrum of solitary fibrous tumors) was included in the differential diagnosis, given its extra-axial origin and potential hypervascularity. However, these tumors typically demonstrate more aggressive imaging features, irregular margins, and bone erosion, rather than hyperostosis. Hemangioblastoma was also contemplated due to the marked cystic component and possible vascular characteristics. Nevertheless, these lesions are most commonly intra-axial and located in the posterior fossa, making this diagnosis less likely in the present supratentorial convexity location. Metastatic disease with cystic degeneration was another consideration. Although cystic morphology may be seen in metastatic lesions, the absence of a known primary malignancy and the presence of a broad dural attachment made this diagnosis less probable. Despite the identification of a dural-based attachment, the large cystic component initially raised concern for a primary intra-axial neoplasm, particularly a high-grade glioma with exophytic growth mimicking an extra-axial lesion. However, several imaging features argued against a primary glial tumor, including the presence of a clear cleavage plane between the lesion and adjacent brain parenchyma, the broad dural attachment, and associated hyperostotic changes of the adjacent skull. Other less likely considerations included an arachnoid cyst with an associated solid component and an atypical epidermoid tumor, although the enhancement pattern was not characteristic of these entities. Parasitic cystic lesions, such as neurocysticercosis or echinococcosis, were also considered in the broader differential of cystic intracranial masses. However, these conditions typically present with multiple cysts, visualization of a scolex, or lack of a solid enhancing mural nodule - features that were absent in this case. Taking all radiological characteristics into account - particularly the extra-axial location, broad dural attachment, capsular enhancement, hyperostosis, and well-defined cleavage plane - the findings were most consistent with a meningioma. Definitive diagnosis was established through histopathological examination.

During the preoperative period, the patient experienced neurological deterioration, characterized by altered consciousness and aphasia. The aphasia was predominantly expressive, suggesting involvement of the dominant frontal opercular region, secondary to mass effect. Medical management of intracranial hypertension with intravenous methylprednisolone (e.g., 120 mg/day) and mannitol (e.g., 20%, 1 g/kg/day in divided doses) resulted in clinical improvement, allowing surgical intervention to proceed. One week after admission, the patient underwent surgery with the aim of complete tumor resection (Simpson grade I resection).

Surgical management

**Figure 3:**
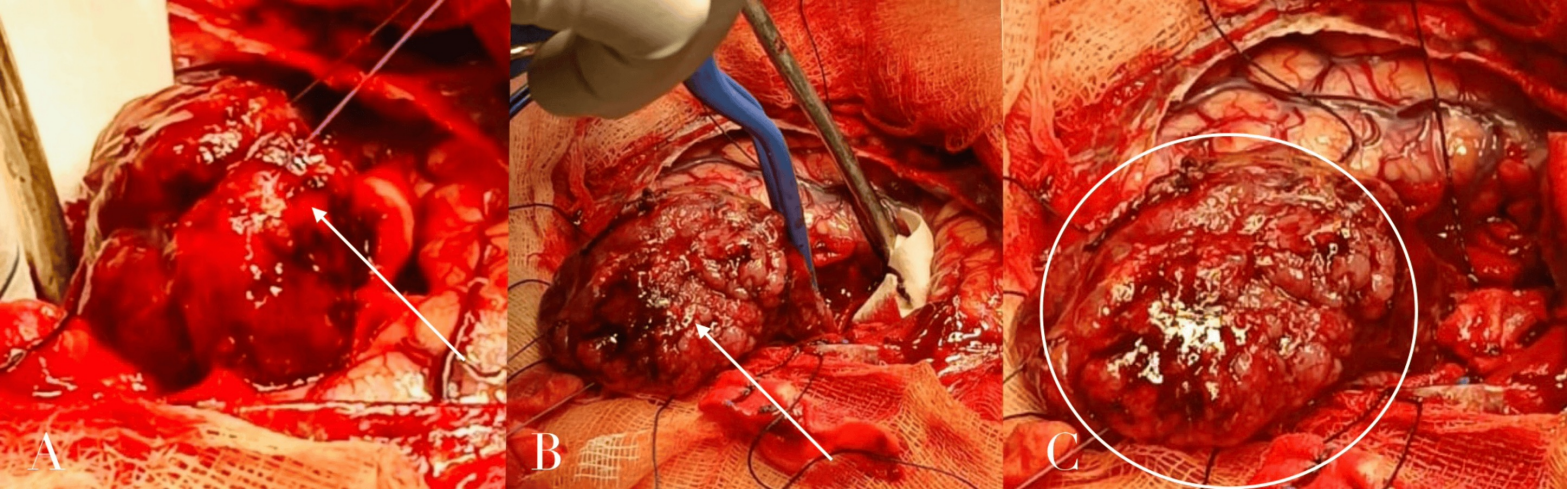
Intraoperative images demonstrating tumor exposure and resection. (A) Initial intraoperative exposure revealing a large, lobulated, extra-axial mass with a reddish, highly vascularized surface (white arrow). (B) Progressive microsurgical debulking of the lesion, showing its nodular architecture and firm consistency (white arrow), with the surrounding brain tissue carefully protected. (C) Circumferential visualization of the tumor (encircled area) prior to further dissection, highlighting its well-defined margins and prominent, vascular surface characteristics.

**Figure 4:**
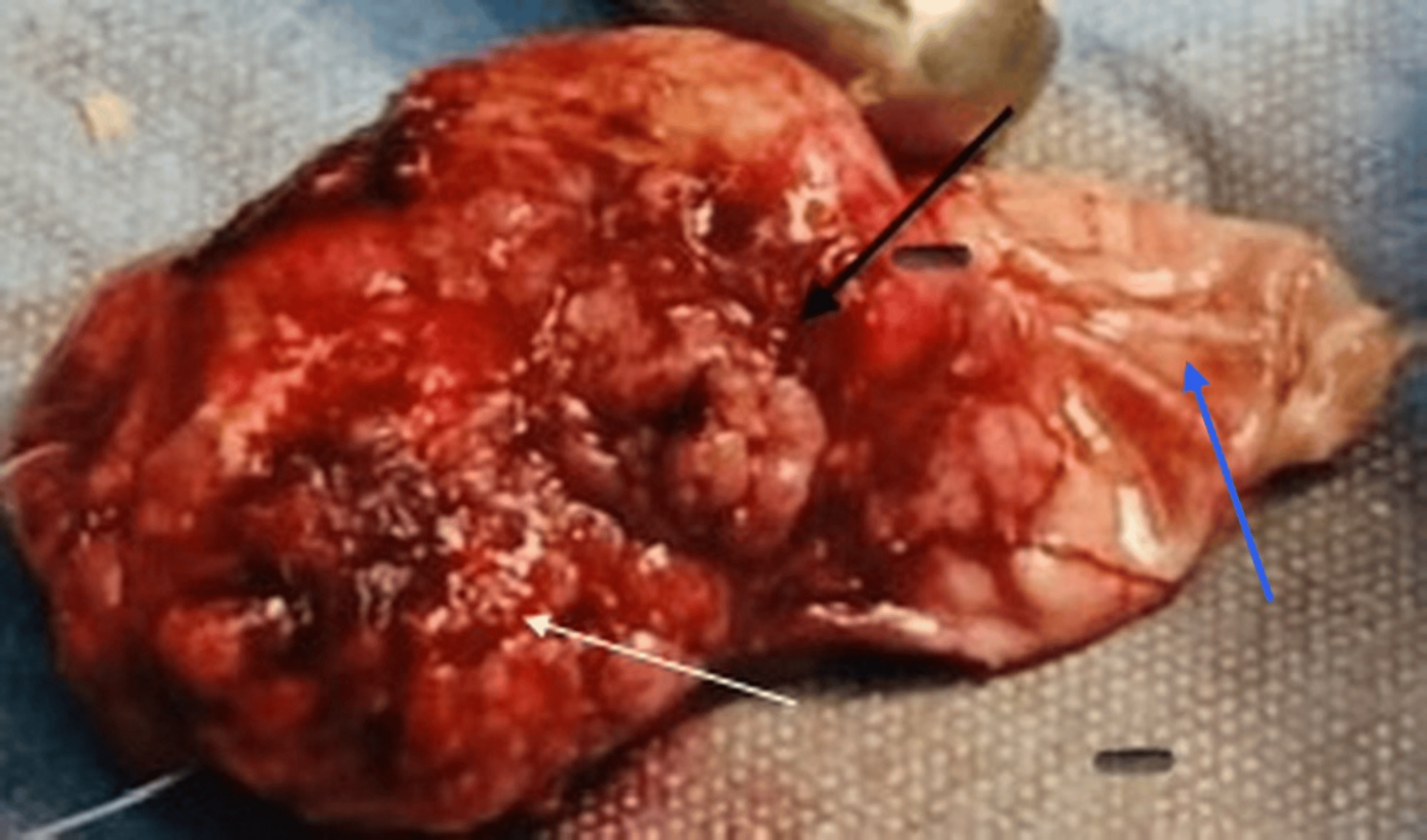
Gross appearance of the tumor specimen. The image demonstrates a well-circumscribed, lobulated mass with a heterogeneous, reddish surface and mixed solid-cystic components. The blue arrow indicates the cystic capsule of the tumor, which appears smooth, translucent, and well-delineated. The black arrow highlights a more solid, nodular portion of the lesion. The white arrow points to areas of surface irregularity with prominent vascularization.

Intraoperatively, hyperostosis of the inner table of the cranial bone flap was observed. A tailored craniotomy, centered over the lesion, was performed based on anatomical and topographical cranial landmarks. The bone flap was removed, and areas of hyperostotic bone were drilled until normal, bleeding cancellous bone was encountered. Initial drainage of the cystic component yielded xanthochromic fluid, contributing to a reduction in intracranial pressure and facilitating surgical exposure. Upon opening the dura mater, a broad dural attachment of the tumor was identified. The dural base was coagulated circumferentially using bipolar cautery to minimize intraoperative bleeding and reduce the risk of recurrence. A complete macroscopic en bloc resection of the tumor was achieved, including both the solid and cystic components, as well as the thick cyst wall. The lesion was carefully dissected along a well-defined cleavage plane between the tumor and the surrounding healthy brain parenchyma. Preservation of cortical veins and meticulous protection of the adjacent cerebral tissue were ensured throughout the procedure. The involved dura was excised, and duraplasty was performed to achieve a watertight closure. Hemostasis was carefully secured prior to replacement of the bone flap and layered wound closure (Figures 3-4).

Histopathological findings

Specimen

Multiple fragments of intracranial tumor tissue, fixed in formalin and stained with hematoxylin and eosin.

Microscopic Description

Histological examination reveals fragments of a well-circumscribed neoplastic lesion, composed of a relatively uniform population of cells arranged in lobules and characteristic whorled patterns. The tumor cells display round-to-oval nuclei with finely dispersed chromatin and scant-to-moderate eosinophilic cytoplasm. Numerous thin-walled blood vessels are present, some showing vascular congestion. Areas of cystic and degenerative change are identified within the tumor. No tumor necrosis is observed. Mitotic figures are rare, and there is no significant nuclear pleomorphism or cytological atypia. In the examined sections, there is no clear evidence of brain parenchymal invasion.

Histopathological Diagnosis

**Figure 5:**
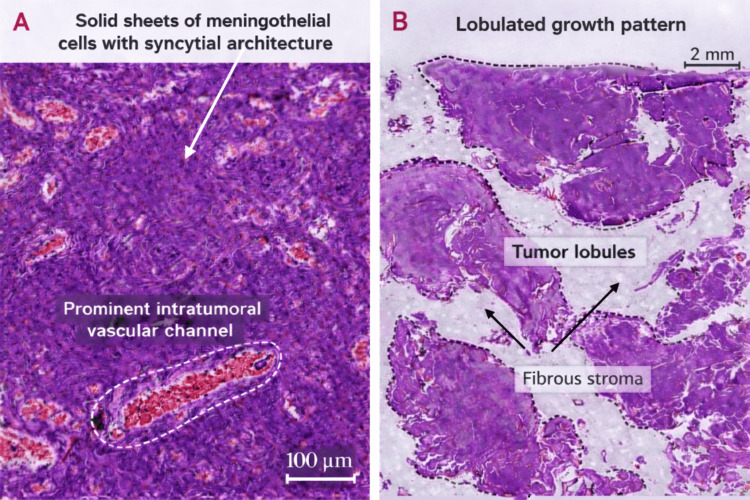
Histopathological examination of biopsy specimen consistent with meningothelial meningioma (hematoxylin and eosin staining). (A) High-power view (scale bar: 100 µm) demonstrating solid sheets of meningothelial cells with syncytial architecture and indistinct cell borders. Prominent intratumoral vascular channels are observed. (B) Low-power view (scale bar: 2 mm) showing a lobulated growth pattern, composed of well-defined tumor lobules separated by collagen-rich stroma.

Meningothelial meningioma (WHO grade I) with associated cystic changes is shown in Figure 5. 

Comment

The histomorphological features are consistent with a benign meningioma, without criteria of atypia or malignancy. The presence of cystic changes represents an uncommon morphological variant and may account for the atypical radiological appearance. Ki-67 (MIB-1) proliferation index and additional immunohistochemical markers were not available in this case, and the diagnosis was established based on characteristic histomorphological findings.

**Figure 6:**
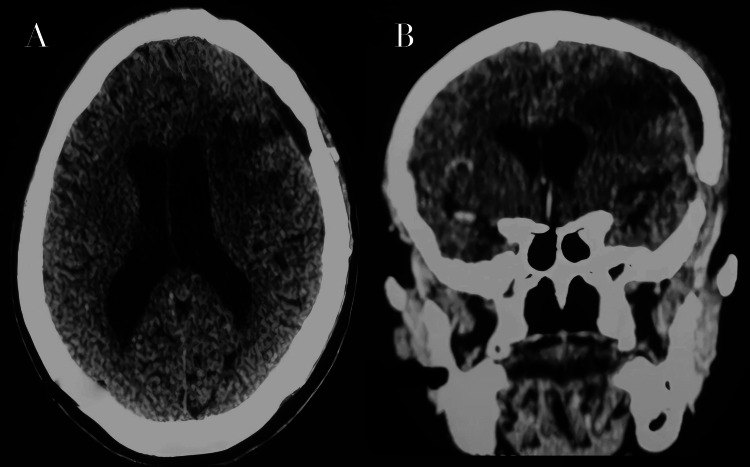
Follow-up contrast-enhanced CT scan performed one month after surgery. Axial (A) and coronal (B) views show postoperative changes without evidence of residual enhancing tumor. CT, computed tomography

The postoperative course was favorable, with progressive recovery of muscle strength and marked improvement in behavioral disturbances and psychomotor slowing, within two months (modified Rankin Scale score: 0-1 [13]). Postoperative neurological examination demonstrated improvement of right-sided motor strength to 4+/5, resolution of aphasia, and normalization of mental status. No new focal deficits were observed. Epileptic seizures remained well controlled under antiepileptic therapy. A follow-up brain scan, performed one month after surgery, showed no evidence of residual tumor (Figure 6).

Ethical approval

According to the regulations of the hosting institution, formal approval from the ethics committee was not required for the publication of this single case report. The patient provided written informed consent, authorizing the use and publication of anonymized clinical data and related images. Every precaution was taken to ensure the patient’s confidentiality and to protect their privacy. No personal identifiers are disclosed in this manuscript.

## Discussion

Meningiomas account for approximately 30%-40% of primary intracranial tumors in adults and arise from arachnoid cap cells. Although classically described as solid, extra-axial masses, cystic meningiomas represent an uncommon morphological variant reported in approximately 4%-7% of all intracranial meningiomas in contemporary cohort studies [7,9]. The presence of a cystic component significantly alters the radiological appearance and may complicate preoperative diagnosis.

The mechanisms underlying cyst formation in meningiomas remain incompletely understood. In the present case, intraoperative findings revealed evacuation of xanthochromic cystic fluid, and histopathological examination demonstrated degenerative cystic changes within the tumor tissue. These findings suggest that the cyst was most likely intratumoral and related to degenerative microcystic changes, rather than reactive peritumoral cyst formation.

The classification proposed by Nauta distinguishes between intratumoral and peritumoral cysts, which carry important surgical implications. Intratumoral cyst walls are typically neoplastic and should be removed to reduce recurrence risk, whereas peritumoral cyst walls may represent reactive gliotic tissue. Importantly, contemporary studies consistently demonstrate that cystic change is not associated with a specific histological subtype, nor with a higher grade according to the WHO classification [7,14]. Most cystic meningiomas remain WHO grade I lesions, supporting their typically benign biological profile.

In the present case, histopathological examination confirmed a meningothelial meningioma (WHO grade I) with degenerative cystic changes - without necrosis, atypia, or increased mitotic activity - supporting a non-aggressive biological profile.

From a clinical standpoint, patients with cystic meningiomas most frequently present with headache, seizures, and focal neurological deficits [9,14]. These manifestations are typically related to mass effect, peritumoral edema, and increased intracranial pressure, rather than intrinsic tumor aggressiveness. Seizures are particularly common in supratentorial convexity lesions and may precede diagnosis by months or even years [11,15]. In some series, a discordance between tumor size and clinical severity has been described, with large cystic lesions occasionally producing relatively mild symptoms - underscoring the heterogeneous behavior of this entity.

In our patient, the initial presentation with tonic-clonic seizures during pregnancy, followed by progressive headache, hemiparesis, expressive aphasia, psychomotor slowing, and urinary incontinence, was consistent with a dominant frontal convexity lesion exerting significant mass effect. The clinical picture was primarily attributable to compression of eloquent cortex and corticospinal pathways, rather than invasive tumor growth. Pregnancy, particularly in the third trimester, induces significant hormonal and hemodynamic changes that may accelerate the growth of hormone-responsive tumors such as meningiomas. Elevated placental growth hormone and insulin-like growth factor-1, together with markedly increased estradiol and progesterone levels, can promote tumor proliferation, angiogenesis, and peritumoral edema. Concurrent plasma volume expansion and increased venous pressure may further enhance tumor swelling. These mechanisms can precipitate rapid mass effect and neurological deterioration, including new-onset seizures, during late gestation [8].

Cystic meningiomas remain a well-recognized diagnostic challenge in neuroimaging [8,14]. The cystic component may be intratumoral or peritumoral and may obscure classic extra-axial features such as broad-based dural attachment, dural tail sign, cerebrospinal fluid cleft, and hyperostosis. Consequently, these lesions are frequently misinterpreted preoperatively as high-grade gliomas, metastatic tumors with cystic degeneration, hemangioblastomas, or pilocytic astrocytomas - particularly when a mural nodule configuration is present [16].

The presence of hyperostosis and a well-defined cleavage plane, as observed intraoperatively in this case, strongly favored the diagnosis of meningioma. Nevertheless, imaging alone remains insufficient for definitive diagnosis, emphasizing the importance of histopathological confirmation [8,9,17].

Surgical resection remains the cornerstone of treatment for symptomatic cystic meningiomas. Contemporary series demonstrate that gross total resection can be achieved in the majority of cases when meticulous microsurgical technique is applied [7,14]. The extent of resection, commonly graded according to the Simpson classification, remains the most significant predictor of recurrence. A Simpson grade I resection - comprising complete tumor removal with excision or coagulation of the dural attachment and treatment of hyperostotic bone - offers the lowest recurrence rates.

In cystic variants, particular attention must be paid to removal of the cyst wall when it is tumor-derived, as incomplete excision may predispose to recurrence [7,11]. Initial decompression of the cystic component, as performed in the present case, may facilitate surgical exposure and reduce intracranial pressure.

Multiple studies confirm that cystic morphology alone does not predict aggressive behavior or increased recurrence risk, and prognosis is primarily determined by histological grade and extent of surgical resection [7,14].

For WHO grade I meningiomas treated with Simpson grade I resection, long-term recurrence rates remain low. Postoperative neurological recovery and seizure control are generally comparable to those observed in non-cystic meningiomas [7,11].

In the present case, the favorable postoperative course - with progressive motor recovery, resolution of aphasia, normalization of mental status, seizure control, and absence of radiological residue - aligns with outcomes reported in contemporary literature.

This case highlights several important considerations: cystic meningioma should be systematically included in the differential diagnosis of extra-axial cystic intracranial lesions; radiological atypia does not necessarily imply higher biological aggressiveness; histopathological evaluation remains the definitive diagnostic modality; and complete surgical resection, including treatment of the dural base and hyperostotic bone, remains the key determinant of long-term outcome.

Taken together, current evidence suggests that cystic meningiomas, although rare and occasionally radiologically misleading, generally demonstrate favorable outcomes when appropriately treated. Awareness of this variant is essential to avoid misdiagnosis and to optimize surgical planning.

## Conclusions

Cystic meningioma is an uncommon morphological variant of intracranial meningiomas and may pose a diagnostic challenge due to its prominent cystic component, which can mimic intra-axial cystic neoplasms. Although several imaging features in this case supported an extra-axial origin, the marked cystic morphology broadened the differential diagnosis. Careful evaluation of radiological signs suggestive of extra-axial location - such as a broad dural attachment, a clear cleavage plane, and associated hyperostosis - is essential to ensure accurate diagnosis and appropriate surgical planning.

Gross total resection, including removal of the solid component and cyst wall when feasible, remains the treatment of choice and is associated with favorable neurological outcomes and low recurrence rates. This case underscores the importance of maintaining cystic meningioma in the differential diagnosis of cystic intracranial lesions and highlights the value of meticulous radiological assessment in guiding optimal management.
